# A review on MCFC matrix: State-of-the-art, degradation mechanisms and technological improvements

**DOI:** 10.1016/j.heliyon.2024.e25847

**Published:** 2024-02-10

**Authors:** Asrar A. Sheikh, Fiammetta R. Bianchi, Dario Bove, Barbara Bosio

**Affiliations:** Department of Civil, Chemical and Environmental Engineering (DICCA), University of Genoa, Via Opera Pia 15, 16145 Genoa, Italy

**Keywords:** Molten carbonate fuel cell, LiAlO_2_ structure instability, Matrix manufacturing, Degradation, LCA studies, Composite electrolyte

## Abstract

Molten Carbonate Fuel Cells (MCFCs) are a promising technology as sustainable power generators as well as CO_2_ selective concentrators for carbon capture applications. Looking at the current cell configuration, several issues, which hinders a stable long-term operation of the system, are still unsettled. According to reference studies, the ceramic matrix is one of the most critical components in view of its high impact on the cell performance since it can influence both the stability and the reaction path. Indeed, it provides the structural support and holds the molten carbonates used as electrolyte, requiring a good mechanical strength despite of a porous structure, a high specific surface area and a sufficient electrolyte wettability to avoid the electrode flooding. The matrix structure, its key-features and degradation issues are discussed starting from the state-of-the-art lithium aluminate LiAlO_2_ usually strengthened with Al based reinforcement agents. Since the achievable performance is strictly dependent on manufacturing, a devoted section focuses on available techniques with a view also of their environmental impacts. Considering a still insufficient performance due to the material structural and chemical instability favoured by stressful working conditions, the electric conductive ceramics are presented as alternative matrixes permitting to increase the cell performance combining oxygen and carbonate ion paths.

## Highlights

• Overview of MCFC matrix manufacturing procedures and their environmental impacts.
• Reinforcing agents and new structures of MCFC matrix.
• Cause-effect list of main aging mechanisms involving MCFC matrix.
• Alternative ion conductive MCFC matrixes for composite electrolytes.

## 1 Introduction

A fuel cell is a power generator reaching a higher efficiency than conventional engines, with lower environmental impacts and fuel demand [1,2]. Molten Carbonate Fuel Cells (MCFCs) are a well-established technology as co-generation units in distributed generation and integrated gasification combined cycle power plants [[3], [4], [5], [6], [7]], using different reformate fuel gases as fuel [8,9]. MCFCs have been proposed as Carbon Capture and Sequestration (CCS) technology showing a CO_2_ capture rate of more than 90% [5,10], treating also very dilute CO_2_ streams (<6 vol%) and producing electricity rather than only consuming energy [11] differently from the other available solutions [12,13]. Indeed, at the cathode (Eq. (1)) the oxygen reduction forms carbonate ions which migrate within the molten salt-based electrolyte to the anode where hydrogen is oxidized releasing CO_2_ (Eq. (2)). Here, MCFCs act as a CO_2_ separator from the cathodic stream containing a CO_2_ low percentage, as in a flue gas, to the anodic stream with a CO_2_ more concentrated composition (Eq. (3)) [6]. Usually downstream of the cells, a two-stage Water Gas Shift (WGS) reactor converts the remaining CO into CO_2_ and a PSA (Pressure Swing Absorption)/membrane system separates CO_2_ from H_2_ in the exhaust stream [14].

(1) $$\frac{1}{2}O_{2}+CO_{2}+2e^{-}\to CO_{3}^{2-}\;\left(\text{cathode}\right)$$

(2) $$H_{2}+CO_{3}^{2-}\to H_{2}O+CO_{2}+2e^{-}\;\left(\text{anode}\right)$$

(3) $$H_{2}+\frac{1}{2}O_{2}+CO_{2}\to H_{2}O+CO_{2}\;\left(\text{global}\right)$$

In the development of MCFC technology many milestones have been achieved, partially overcoming design problems. The optimization of microstructural parameters by the introduction of bi-layered electrodes and devoted supports have led to both the power density increase and the working temperature decrease without changing used materials [15,16]. Moreover, modelling aided at a proper cell thermal [17] and mass [6] management as a function of the system operation (i.e., power generation and CCS) by discriminating fluidic, thermodynamic and kinetic effects, which permits to avoid the hot-spot development and to know the exhausted gas composition. Several promising prototypes have been produced in Europe, some megawatt scale plants have also been started-up [18]. The worldwide installed capacity exceeds the 300 MW mainly distributed between USA, with more than 50 active or planned stationary plants, and South Korea with a 59 MW power plant operating for different years [[19], [20], [21], [22]]. Nevertheless, these results are not sufficient for a MCFC competitive commercialization and here the effective application fields are still limited, since the cell lifetime is characterized by a voltage decay rate higher than 10% [23] and the conversion efficiencies are too low reaching around 57% only under a stressful pressured operation [24].

In such a framework, this review aims at a clear thorough discussion on useable ceramic matrixes which have to retain the molten carbonate electrolyte and provide the mechanical strength, resulting one of the most critical cell components. Starting from low number previous references due to the specificity of the topic [25,26], the analysis presents the state-of-the-the-art design (i.e., lithium aluminate planar configuration) dealing with its main properties and issues dependent on both manufacturing and applied working conditions. Opening new perspectives with respect to the preceding literature, the most suitable proposed alternatives in terms of geometry and materials are also discussed. Despite of their low technology readiness level if applied to the molten carbonate cell technology, they result quite promising to satisfy new rising industrial requirements such as ecofriendly manufacturing, easy stack connection and high conductivity at mild temperatures slowing the degradation. In details, manufacturing techniques are illustrated from powder preparation to final cell assembling for planar and tubular configurations with a focus on environmental issues. The lithium aluminate matrix structure is presented by discussing the used reinforcing agents and possible aging effects resulting a lengthily tested material. Reporting more recent research works on this field, the observed degradation processes are presented from microstructural variations to consequent cell performance losses with a clear cause-effect explanation. Overcoming the inert matrix paradigm, its direct involvement into the electrochemical process is explained for the composite electrolyte operation where the matrix retains the conductive molten carbonate electrolyte as well as contributes to the ionic charge migration.

## 2 MCFC matrix: features and manufacturing

Referring to the conventional cell structure, the matrix serves as (i) electron insulation, (ii) gas barrier between the anode and the cathode and (iii) support for the molten electrolytes, which are retained in its porous structure allowing the ion migration inside [27]. The electrolytes are usually alkaline salts [1], such as lithium and potassium carbonates, molten at MCFC operating temperatures [28]. A sufficient capillary force for their uptake and retention inside the matrix is obtained by keeping the pore diameters of the matrix smaller than those of the electrodes [26,29] and by having a good wettability of the electrolyte melt [30]. The optimal management is obtained when the electrolyte partially fills the electrode pores and completely fills the matrix ones. Generally, the electrolyte content within the matrix has to be maintained greater than 50 vol% [30], compared to a maximum of 30 vol% within the electrodes with usually a smaller value at the cathode showing an operation more sensitive to the electrolyte content [31,32]. Referring to the cell pore to filling ratio, defined as the electrolyte volume over the total pore volume, minimal polarization resistances have been reported at 50–80% with an exponential increase for lower and higher values [33]. Here acceptable cell behaviors are usually obtained if the matrix has a porosity between 50 and 70% [27,29,34] and the pore size is less than 1 μm [26] with an average pore size optimal distribution of 0.1–0.3 μm [29,33]. A higher number of pores would increase the ionic conductivity but make the matrix more fragile causing its premature degradation [28]. Otherwise, by keeping the pore size small and so penalizing the cell electrochemical performance, a strong matrix could be manufactured with a low risk of crack formation resulting more stable also under high pressure gradients at its two sides in agreement with Young-Laplace equation (**Eq. 4**) [28].

(4) $$\Delta P=\frac{\gamma \;\cos\;\theta }{D}$$

Where *Δp* is the differential pressure between the anode and the cathode (N/m^2^), *γ* the coefficient of surface tension of the electrolyte (N/m^1^), *θ* the contact angle between the matrix and the electrolyte (−), *D* the pore diameter (m).

An appropriate porosity range to obtain a high electrical conductivity can be evaluated by the Meredith–Tobias equation as follows (Eq. (5)) [35]:

(5) $$\rho =\rho_{0}\delta^{-2}$$

Where *ρ* is the specific resistance of the matrix (Ωm), *ρ*_*0*_ the specific resistance of the electrolyte (Ωm) and *δ* is the matrix porosity (−).

Another important characteristic of the matrix is the particle Specific Surface Area (SSA) that affects greatly the mean pore size and the porosity [33]. A specific surface area of ∼10 m^2^/g is usually considered for MCFC applications [1,33], however high-performing matrixes can reach up to ∼12 m^2^/g [28].

Since the retained alkaline based carbonate electrolytes (i.e., Li_2_CO_3_/K_2_CO_3_/Na_2_CO_3_) are very reactive, the matrix has to be inert and chemically stable to guarantee a cell long-term operation [36]. Moreover, MCFC matrix mechanical strength should reach at least 90–100 gf/mm^2^ [37].

**Fig. 1:**
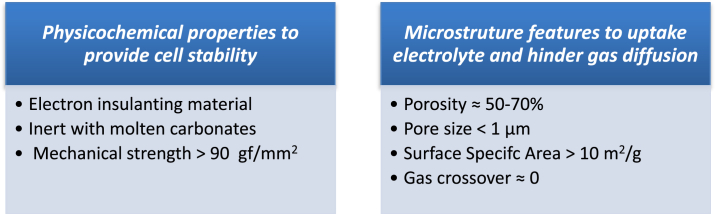
MCFC matrix target performance and microstructural features.

Fulfilling most these criteria (Fig. 1), the state-of-the-art material consists of lithium aluminate LiAlO_2_ reinforced by Al agents. Among three allotropic forms, ***α***-LiAlO_2_ hexagonal structure, β-LiAlO_2_ monoclinic structure and ***γ***-LiAlO_2_ tetragonal structure [38], α-phase [[39], [40], [41]] and ***γ***-phase [28,29,33,40,42] are mainly used as MCFC matrices.

### 2.1 Fabrication techniques

There are two possible MCFC configurations: the most common planar design [43,44] and a rarer tubular structure [45,46]. In the first case the cells are electrically connected in series, overlapped one another until reaching the desired power size. Whereas in the tubular configuration the cells are electrically connected in parallel, here each unit is not influenced by the conductivity of the adjacent ones. Nevertheless, a good adhesion between electrodes and electrolyte is quite challenging avoiding excessive contact resistances as well as guaranteeing the electrolyte retention within porous structures.

Referring to the planar matrix, the green sheets are usually fabricated by the tape casting of a slurry produced by mixing the raw material powders (LiAlO_2_) with binders, plasticizers, dispersants and defoamers within a solvent in well-defined compositions and adding a reinforcement [26,29,33,36,40,41]. Their function is specified in Table 1.

**Table 1:** Slurry components and their functions [33,41].

Component	Function
Raw material	Basic component of matrix structure (usually lithium aluminate powders)
Solvent	Mix homogenously the raw material with other components
Binder	Increase the matrix strength and toughness
Plasticizer	i) Reduce the plastic limit temperature of the binder below room temperature resulting in a good fluidity and avoiding its condensation under atmospheric conditions ii) Reduce the viscosity and increase the flexibility of LiAlO_2_
Dispersant	i) Prevent the powder particles from agglomerating ii) Reduce the slurry viscosity and improve its rheology mainly by increasing the repulsive potential energy and by lowering the gravitational potential energy among particles
Defoamer	Enlarge the air bubbles in the slurry to the extent of rupture in the milling process
Reinforcement	Increase the matrix mechanical strength and integrity with other components

Referring to LiAlO_2_ manufacturing, the powder formation derives from several processes: (i) the solid-state method, (ii) the co-precipitation, (iii) the molten salt hydrolysis, (iv) the sol-gel method and (v) the combustion synthesis. Using commonly Al_2_O_3_ and Li_2_CO_3_ as precursors and an operating temperature also higher than 1000 °C, the solid-state method is the most frequent and the simplest [47]. Nevertheless, there are several issues such as particle agglomeration, influence of the used reactants on the final product particle size and Li partial loss by evaporation due to high temperatures. Co-precipitation with an aqueous surfactant solution is another valid technique for a large-scale production, requiring inorganic metal salts and mild working conditions [48]. The molten salt hydrolysis is a less common approach producing a low purity LiAlO_2_ [46]. The sol-gel method is based on the hydrolysis of lithium and aluminum alkoxides followed by a heat treatment at ∼500 °C leading to high purity powder generation [49]. Despite of the benefit of lower temperatures, the particle morphology is not easy to monitor due to the precursor instability and common reactants are quite expensive. Finally, the combustion synthesis method usually applies an aqueous solution of glycine–urea and metal nitrates as the precursors to obtain homogeneous and fine particles. Nevertheless, there is the risk of amorphous carbon-containing compound formation in case of an incomplete combustion [50]. An alternative involves the use of Al_2_O_3_ and LiOH as the precursors and urea as the fuel reaching high purity powders without nitrates [51].

Since Al_2_O_3_ is an expensive precursor, alternatively the boehmite (AlOOH·nH_2_0) can be mixed with LiOH in water, which makes the synthesis procedure low-cost, energy saving and environmentally friendly since the boehmite is a quite cheap material with a high dispersion in water [46]. It has been also investigated ***α***-LiAlO_2_ powder synthesis using industrial grade Al(OH)_3_ and Li_2_CO_3_ as precursors which permit to obtain a matrix with a high mechanical strength [52].

The standard manufacturing technique for MCFC planar cells consists of the tape casting resulting a preferable procedure over the cold pressing [40], in view of its reproducibility and easy scale-up [28]. The slurry is usually prepared by mixing LiAlO_2_ powders in an organic solvent since LiAlO_2_ hydrolyzes into LiOH.H_2_O and Al(OH)_3_ in water [33,41]. LiAlO_2_ powder specific surface area as well as its weight content in the initial slurry are properly selected to optimize the final microstructure. The ball milling reduces the particle size until reaching a uniform slurry with the desired Particle Size Distribution (PSD) and without agglomerates [28]. Small particles allow a high SSA, however too fine elements can generate a matrix with a poor strength. Nevertheless, nano-LiAlO_2_ matrix has been also reported resulting in a nano-size material (15–26 nm) with a uniform distribution and a stable microstructure [53]. Referring to the mass fraction of LiAlO_2_ powder, a low mass content is good for the slurry rheological properties but very low values can reduce the collision frequency of the powder particles with the milling media thereby decreasing the possibility of the required PSD achievement [41]. Here, after introducing gradually other components into the slurry, the ball milling is followed by the filtering, the de-gassing, the tape casting, and the drying for solvent evaporation [29,41]. Matrix green sheets are usually treated at high temperatures (around 650 °C) to burn out binders and other additives finalizing the final microstructure [28,29]. After sintering the solid matrix, the molten electrolyte can fill the created porosities by infiltration technique. However, solid carbonates can be directly mixed within the initial slurry to optimize the electrolyte amount and reduce the possible leaks. Following the main benefits are reported: i) improvements in mass production process, ii) uniformly distributed electrolytes within the matrix, iii) a stable stack height, iv) absence of mechanical stresses during the electrolyte penetration inside pores by capillary forces and v) a reduction of contact resistances between molten electrolytes and LiAlO_2_ particles [54].

An alternative, just validated at lab scale, is the colloidal crystal templating allowing for the direct production of a LiAlO_2_ porous layer suitable as MCFC matrix. It consists of filling the voids of close-packed arrays of monodisperse polymeric spheres, such as polystyrene and poly methyl methacrylate, with inorganic precursors. The templating spheres are then removed by calcination, extraction or etching [55].

Considering the tubular design manufacturing, a punching pipe is used as the support which the cathodic slurry is coated and sintered on. Then the matrix slurry, prepared following the same procedure of planar cells, is dried after layering on the previously sintered cathodic layer. After drying the sample, the anodic Ni powder slurry is applied and the whole unit is finally sintered. During the sintering Ni particle joining and anode shrinkage produce a fastening force on the matrix which improves the contact among layers in addition to apply a hose band outside. Finally, carbonates are impregnated on both cathode and anode sides (i.e., inside and outside the pipe) [44].

Looking at environmental impacts, the cell manufacturing has been pointed as one of the most critical steps in MCFC lifecycle [[56], [57], [58]]. Indeed, severe issues are correlated to the lithium aluminate production and the solvent toxicity. Focusing on the matrix, the environmental impacts in terms of abiotic resource depletion, acidification, eutrophication and global warming have been estimated as 10.0%, 5.7%, 6.1% and 3.5%, respectively, of the whole cell manufacturing [59]. Table 2 summarizes the main results derived from published LCA studies on matrix fabrication, pointing out the electricity consumption and the possible polluting products.

**Table 2:** LCA studies on the matrix fabrication.

Reference	Electrical Energy (kWh/kW_MCFC_)	CO_2_ (kg/kW_MCFC_)	CO (g/kW_MCFC_)	NO_x_ (g/kW_MCFC_)	SO_2_ (g/kW_MCFC_)	CH_4_ (g/kW_MCFC_)	NMVOC (g/kW_MCFC_)	BENZENE (g/kW_MCFC_)
[60,61]	72.83	127.00	25.20	441.00	1.50	36.20	16.80	0.03
[62,63]	39.30	0.47^b^	0.09^b^	0.31^b^	0.91^b^	0.02^b^	0.04^b^	–
	1.93^a^							

(a) Starting from raw materials.

(b) Starting from slurry preparation.

An alternative method is the aqueous tape casting permitting the impact reduction correlated to the organic solvent (toluene or isopropyl alcohol) use in the slurry preparation [33]. Nevertheless, under such conditions LiAlO_2_ can react with water producing low quantities of hydrated phases which affect the matrix structural and thermal stability [64]. In addition to this undesired by-product formation, the aqueous solution is also characterized by a lower wettability and a higher viscosity resulting less suitable than the organic solvents, which remain the best choice in view also of a smaller drying time due to a higher volatility [41]. LiOH·H_2_O and Al(OH)_3_ can be added as Li–Al precursors to γ-LiAlO_2_ in order to prevent the hydrolysis, reinforce the matrix by the following synthesis reaction (Eq. (6)) and avoid an excessive agglomeration of LiAlO_2_ powders [33].

(6) *LiOH.H*_*2*_*O + Al(OH)*_*3*_ → *LiAlO*_*2*_*+ 3H*_*2*_*O*

As an alternative to the tape casting, the plastic extrusion results a quite effective eco-friendly technique without requiring a solvent use. Indeed, this process prepares a mixture of just ceramic powders and binder, which is then subjected to the extrusion producing thin sheets (0.25–0.75 mm) [65].

Table 3 summarizes reference proposed manufacturing processes focusing on the slurry composition.

**Table 3:** Characteristics of slurry constituents.

Reference	Technique	Raw material	Solvent	Binder	Plasticizer	Dispersant	Defoamer	Reinforcement
[26]	Tape casting + hot pressing	LiAlO_2_ (36.3 wt%)	Toluene + Ethanol (47.2 wt%)	Polyvinyl butyral (9.1 wt%)	Dibutyl phthalate (5.8 wt%)	DisperBYK-110 (1.1 wt%)	SN D-348 (0.5 wt%)	Metal wire mesh
[28]	Tape casting	γ-LiAlO_2_	Ethanol	Polyvinyl Butyral	Dibutyl phthalate	Solsperse-20000	Agitan DF300 M	–
[29]	Tape casting	γ-Al_2_O_3_ + LiOH + NaOH (30.2 wt%)	Toluene + Ethanol (52.7 wt%)	Polyvinyl butyral (7.5 wt%)	Dibutyl phthalate (9.0 wt%)	Solsperse-9000 (0.6 wt%)	–	γ-Al_2_O_3_ rod shaped particles
[33]	Aqueous tape casting	γ-LiAlO_2_ + LiOH.H_2_O + Al(OH)_3_ (59.5 wt%)	De-ionized water (28.4 wt%)	Protein (Ovalbumin or gelatin) (6.3 wt%)	Glycerin (5.4 wt%)	Darvan-C	(0.4 wt%)	–
[36]	Tape casting	γ-LiAlO_2_ + additives (36.3 wt%)	Butanol + Isopropanol (47.2 wt%)	Polyvinyl butyral (9.1 wt%)	Polyethylene glycol (5.8 wt%)	Zetasperse, (1.1 wt%)	Surfynol (0.5 wt%)	Al powders, ***α***-Al_2_O_3_ fibers + Li_2_CO_3_
[38]	Tape casting	***α***-LiAlO_2_+ additives (36.3 wt%)	Toluene + Ethanol (47.2 wt%)	Polyvinyl butyral (9.1 wt%)	Dibutyl phthalate (5.8 wt%)	DisperBYK-110 (1.1 wt%)	SN D-348 (0.5 wt%)	Al powder + Li_2_CO_3_
[40]	Tape casting	γ-LiAlO_2_+ additives (38.8 wt%)	Ethanol (38.4 wt%)	Polyvinyl butyral (9.7 wt%)	Dibutyl phthalate (11.6 wt%)	Solsperse-20000 (0.75 wt%)	SN D-348 (0.75 wt%)	Al powder + Li_2_CO_3_
[41]	Tape casting	***α***-LiAlO_2_ + γ-LiAlO_2_ + additives (40.1 wt%)	Cyclohexanone + Butyl alcohol (36.0 wt%)	Polyvinyl butyral (13.0 wt%)	Polyethylene glycol (9.0 wt%)	Triolein (1.3 wt%)	Silicone oil (0.6 wt%)	Al_2_O_3_ fiber
[53]	Tape casting	***α***-LiAlO_2_ (22.4 wt%)	Ethanol (62.8 wt%)	Polyvinyl butyral (6.2 wt%)	Dibutyl phthalate (7.4 wt%)	DisperBYK-110 (0.6 wt%)	SN D-348 (0.6 wt%)	Electrolyte particles within matrix slurry

## 3 MCFC matrix: state-of-the-art

The allotropic forms of lithium aluminate which satisfy MCFC matrix requirements are ***α***- and ***γ***-LiAlO_2_. However, the material stability strictly depends on the working environment which can lead to the phase transition phenomena, justifying the proposal of both solutions in literature as a function of temperatures and CO_2_ compositions. In early studies the matrix had to be primarily compatible with used electrolytes; here ***γ***-LiAlO_2_ was initially applied showing a high corrosion resistance to molten carbonates [37]. Moreover, from the thermodynamic phase diagram under air, ***γ***-LiAlO_2_ should have the most stable crystal structure at high working temperatures (i.e., 650–750 °C) [66]. Nevertheless, the fuel cell real conditions with CO_2_–H_2_ rich atmospheres vary the material behavior [67]. Indeed, the presence of an acidic or a reduced environment can drive LiAlO_2_ phase transformation [37]. ***α***-LiAlO_2_ exhibits a higher stability under MCFC operation [22,68]; indeed, it remains stable in both H_2_/CO_2_ and air/CO_2_ at 650 °C, differently from ***γ***-LiAlO_2_ which converts to ***α***-LiAlO_2_ under a H_2_/CO_2_ feed. Nevertheless, ***α***-LiAlO_2_ can transform into ***γ***-LiAlO_2_ in H_2_ pure atmosphere (however a rare MCFC working condition) due to the energetically unfavorable adsorption of the hydrogen on its facets [69]. If on the one hand ***α***-phase has a higher microstructural stability, on the other it is not superior to ***γ***-phase in terms of the mechanical strength requiring a reinforcement as well [40,70]. Before discussing the possible solutions to improve mechanical properties in the following section, Table 4 summarizes the main benefits and drawbacks of both allotropic forms.

**Table 4:** α-LiAlO_2_ vs. γ-LiAlO_2_: features and issues.

Feature	***α***-LiAlO_2_	***γ***-LiAlO_2_
Stability in air	High at low temperatures (<∼650 °C)	High at high temperature (>∼650 °C)
Stability in air/CO_2_ @(650–750 °C)	No phase transition	No phase transition
Stability in H_2_/CO_2_ @(650–700 °C)	No phase transition	***γ*** to ***α*** transition
Stability in 100% H_2_ @(650 °C)	***α*** to ***γ*** transition	No phase transition
Stability under thermal cycling	Good	Poor
Structural stability	Requires reinforcement	Requires reinforcement

### 3.1 Mechanical issues and reinforcement agents

**Fig. 2:**
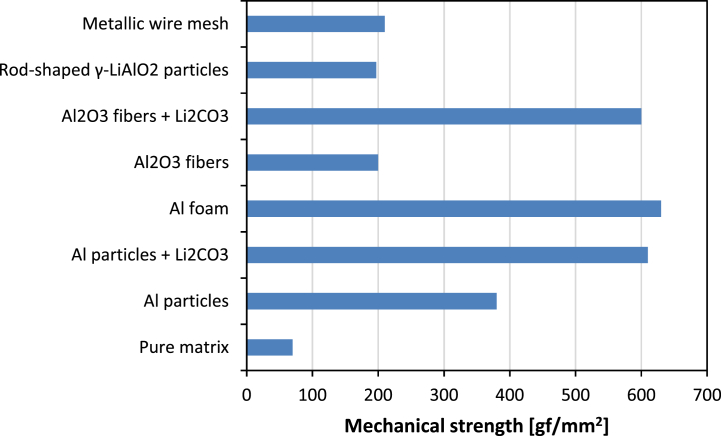
Improvements of the matrix mechanical strength by the introduction of reinforcing agents considering the pure matrix value [36] and its increase in case of Al particles [36], Al particles + Li_2_CO_3_ [36], Al foam [69], Al_2_O_3_ fibers [36], Al_2_O_3_ fibers + Li_2_CO_3_ [36], rod-shaped γ-Li_2_CO_3_ particles [29] and metallic wire mesh [26].

The microcrack formation and propagation easily develop in a pure brittle ceramic matrix due to thermal and mechanical stresses encountering during the cell startup and operation [25]. Crack sources have been evaluated throughout the manufacturing due to the weak bonding of the ceramic particles and the impregnated carbonates in un-sintered matrixes [33,40]. Mechanical stresses increase also after the removal of binders during the startup heating. During cooling the mismatch between the thermal expansion coefficients of LiAlO_2_ (∼10 nm/°C) and of carbonate-based electrolytes (∼20 nm/°C) can generate compressive stresses on the matrix particles and tensile stresses on the carbonates which exceed the material mechanical resistances [71]. The addition of reinforcing elements permits to overcome these issues and increase the mechanical strength by improving the structural stability and hindering the crack propagation. Several alternatives have been proposed starting from metal and ceramic particles in different shapes and amounts [36,[38], [39], [40]] to metallic mesh integration [26], Al_2_O_3_ and LiAlO_2_ fibers [36,41] and more recently high performing Al foam based matrix [69,72], looking for a compromise between an effective increase of the mechanical strength and the hindering of unwanted degradation processes due to the addition of further elements within the matrix. Fig. 2 reports reference materials with their corresponding mechanical property.

The first widely used reinforcing agents have been the metal particles, since they allow for improving several properties of the matrix, such as the stiffness, the thermal behavior and the resistance to abrasion. Acting as “crack bowing” or “crack deflection”, they reduce the stress intensity, obstruct the crack front and hinder it from propagating [40]. Zn and, above all, Al have been used as the dispersed metal powder phase to increase the bonding between matrix and electrolyte as well as to reduce the thermal expansion coefficient mismatch. For instance, Al is characterized by a higher value than lithium aluminate one (23 nm/°C vs. 10 nm/°C), here the matrix thermal expansion coefficient improves with Al content increase [40]. In addition to a higher mechanical strength, the fully assembled cells are more stable under thermal cycles and long-term operation. Looking at the manufacturing procedure, these reinforcements have low melting points (∼660 °C and ∼419 °C for Al and Zn respectively) and can sinter as metal-metal or metal-ceramic particles during both startup and operation [25]. According to a U.S. patent [73], the suggested amount of Al as the reinforcement material for MCFC matrix is ∼3–45 vol% with a particle size distribution of ∼0.1–20 μm. Indeed, the mean pore size of the matrix increases by increasing Al particle size, while the porosity is mainly influenced by the amount of Al particles [38]. When Al particle content is high, the porosity decreases due to a more probable Al particle sintering. Moreover, Al at high percentages can react with the molten carbonates hosted in the matrix causing degradation issues as better discussed below. According to experimental observations, the matrix mechanical strength increases almost linearly for an Al content of 0–30 wt% and a particle size of ∼3 μm, but for large particles (>∼30 μm) the reinforcing effect is much smaller due to Al particle sintering.

As an alternative, rod/needle shaped ***γ***-LiAlO_2_ particles can be used to prevent the crack propagation. Their manufacturing is easy, and they can be uniformly dispersed within the matrix even by tape casting. Nevertheless, the pore forming agents such as carbon powders and ammonium carbonates have to be added into the slurry in order to reduce ***γ***-LiAlO_2_ particle synthesis time and avoid by-product formation during manufacturing [74]. Rod shaped particle addition decreases the porosity and increases the pore size compared to the pure matrix values (however remaining in acceptable ranges) but also increases the flexural strength [29,33].

Different from the previous cases, a metallic mesh has been also applied on ceramic materials. The matrix is three time stronger than the pure structure by introducing a stainless-steel wire mesh (AISI 304), but the cell has a significant internal resistance offered by this [26]. Here, the use of a thinner mesh and/or the elimination of its bumping parts are some requested changes to take advantages from this more resistant structure without a significant performance decay.

Knowing the benefits of fibers as material strengthening such as in case of concrete and cements, ***α***-Al_2_O_3_ and ***γ***-LiAlO_2_ fiber application has been reported useful in reducing the matrix cracking and providing a high surface area over long term cell operation. These micro-fibers strengthen the microstructure by attracting the cracks towards them due to the shear resistance at fiber-matrix interface [25]. Initially, ceramic fibers were mainly composed by the same material of the matrix obtaining a higher flexural strength (more than 20–40 %) and corrosion resistance [75] as well as a superior cell stability [76]. More recently, Al_2_O_3_ fiber use has permitted to reach a three times higher mechanical strength with respect the pure matrix value [36,41]. Despite this competitive performance, Al particles are still considered as a better reinforcing medium over fibers due to i) an “easier addition” since Al_2_O_3_ fibers are commonly synthesized from a mercury-based solution and ii) a lower required carbonate amount to achieve the same cell performance.

A more recent solution consists of Al foam use [69], which forms an alumina skin layer during the cell operation. Indeed, this layer reacts with Li_2_CO_3_ based electrolyte to form LiAlO_2_ favoring the matrix strength. During sample manufacturing the Al foam has to be first pre-oxidized at 800 °C at least to avoid any deformation by Al melting in the cell startup and to have a good wetting of the matrix for the electrolyte. An ex-situ oxidation at higher temperatures can be also performed to guarantee that an enough volume of Al-foam is oxidized prior to the fabrication of Al-foam reinforced matrixes [71]. The reinforced matrix has up to a 9.4 times higher mechanical strength and a ∼50% lower pore size compared to the pure matrix with a comparatively narrow pore size distribution allowing for a greater capillary action and molten electrolyte retention.

It is noteworthy that the effectiveness of reinforcements can strictly depend on the sintering procedure. Here the sintering aids can be used to improve the mechanical strength by favoring the bonding among particles. In this regard, the addition of B_2_O_3_ to the conventional LiAlO_2_ matrix allows a significant enhancement due to Li_2_AlBO_4_ new phase formation [77]. Aluminum acetylacetonate is another potential candidate that forms LiAlO_2_ necks among matrix particles by reacting with Li_2_CO_3_ [78].

Referring to previously cited solutions, several samples have been tested by evaluating their effectiveness in term of (i) the microstructural features, (ii) the mechanical strength, (iii) the chemical stability and (iv) the durability (Table 5). The first two points are commonly estimated on just the specific matrix layer directly by computing the three-point bending strength [33] or indirectly detecting electrolyte leaks due to matrix crack through differential pressure tests [79]. Whereas the chemical stability and the durability are usually evaluated on fully assembled cell samples under a nominal MCFC operation referring to both the electrochemical characterization which monitors the time evolution of internal resistance, Open Circuit Voltage (OCV) and cell voltage under load, and the microstructural analysis [28,40,53,80].

**Table 5:** State-of-the-art LiAlO_2_ features in terms of microstructure, mechanical stress and durability depending on performed characterization techniques.

Reference	Porosity (%)	Pore diameter (μm)	Mechanical stress (gf/mm^2^)	Stable operation	Matrix property characterization	Cell electrochemical characterization
[26]	53	0.26	–	>1500 h	Hg porosimetry, SEM analysis and gas chromatography	Time evolution of resistance, OCV and cell voltage (@ 150 mA/cm^2^)
[28]	69.4	<1	–	1000 h	SEM analysis, BET surface analysis	Time evolution of resistance and maximum power density
[29]	55	0.05–0.30	197	–	Hg porosimetry, SEM analysis, XRD, 3-pt bending strength test	–
[33]	50–65	0.15	190	–	Hg porosimetry, SEM analysis, XRD, 3-pt bending strength test	–
[36]	66	<1	610	–	SEM, XRD, 3-pt bending strength test	–
[38]	50	0.14	200	1250 h	Hg porosimetry, SEM analysis, 3-pt bending strength test, gas chromatography	Time evolution of resistance, OCV and cell voltage (@ 50-100-150 mA/cm^2^)
[40]	61	0.10	306	15 thermal cycles	Hg porosimetry, SEM analysis, 3-pt bending strength test, Dilatometry	Evolution of OCV and cell voltage (@ 150 mA/cm^2^) due to thermal cycles
[41]	50	0.10–0.40	–	1500 h	SEM analysis, Laser particle analysis, BET surface analysis	Time evolution of cell voltage (@ 200 mA/cm^2^)
[53]	63	0.10–0.30	–	18 thermal cycles	Hg porosimetry, SEM analysis, EDX	Time evolution of OCV and cell voltage (@ 150 mA/cm^2^) due to thermal cycles

## 4 MCFC matrix: degradation mechanisms

**Fig. 3:**
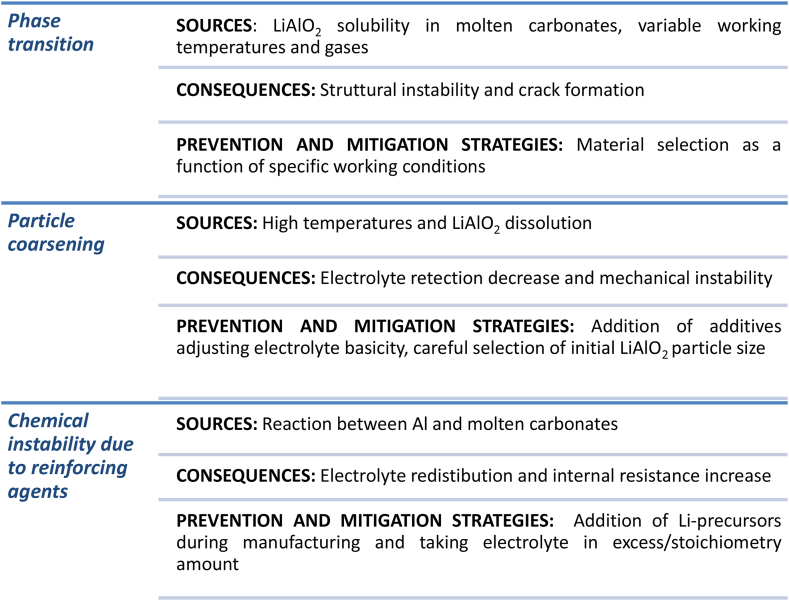
Overall degradation processes involving MCFC matrix.

Matrix degradation is one the main issues concerning lithium aluminate use, as confirmed by several experimental findings [22,37,[81], [82], [83]]. Material instabilities are commonly evaluated during long-term working due to the particle growth and the phase transitions above all, in addition to the composition changes caused by the reinforcing agent addition (Fig. 3).

### 4.1 Dissolution and phase transition

One of the main LiAlO_2_ drawbacks is its tendency to dissolve into carbonates [25], which triggers several other microstructural changes, such as the particle growth and the phase transition depending on LiAlO_2_ solubility in the molten carbonates [81], as well as the LiAlO_2_ diffusion and precipitation [80]. The solubility is influenced by working conditions in terms of temperature, exposed atmosphere (i.e., air/CO_2_/H_2_) and carbonate composition to a lesser extent. Referring to MCFC common working conditions, there is a higher dissolution at temperature increase and CO_2_ partial pressure decrease [81].

Considering three LiAlO_2_ allotropic forms under air, β-phase has always the highest solubility indicating its instability at any temperature. At around 650 °C the dissolution constant of ***γ***-configuration is higher than ***α***-configuration one (i.e., more stable ***α***-structure), whereas at 700 °C the opposite trend is evaluated (i.e., more stable ***γ***-structure) [80]. Since the dissolution is usually followed by the phase transition to create a more stable structure, the temperature increase means ***γ***-phase formation. In addition to the working temperatures, the exposition time also has a significant influence: the complete phase conversion usually requires several thousands of hours [84], nevertheless a difference of few testing hours can result in different material final structures at temperatures intermediate between ***α***- and ***γ***-phase domain [66]. Effects due to the gas exposition have been also demonstrated and correlated to the particle growth in Ref. [37], since highly soluble samples show a greater particle coarsening. Matrixes consisting of nano-sized ***α***-LiAlO_2_ particles were exposed to H_2_–H_2_O–N_2_, N_2_, H_2_–CO_2_–N_2_, and air-CO_2_ atmospheres at 650–750 °C for 100–500 h. The particles with an initial structure of 20 nm grow largely in cases without CO_2_ at any testing temperature. At 750 °C the phase transition also occurs reaching 97–99 mol% of ***γ***-phase when ***α***-LiAlO_2_ crystal size exceeds 150–200 nm after about 100 h. The critical crystallite size of ***α***-LiAlO_2_ before a prominent phase transition is reported at ∼30 nm, excluding the case under N_2_ atmosphere where a small phase transition is detected below. The particle growth remains under the critical crystallite size in a CO_2_ containing atmosphere at both 650 °C and 750 °C after 500 h without here observing a phase transition. Effects of CO_2_ partial pressure on LiAlO_2_ phase evolution is further explored thoroughly in Ref. [82], by conducting the solubility tests for micro-sized ***α***-LiAlO_2_ at 650 °C for 100–400 h under three CO_2_ atmospheres (0.3 atm, 0.005 atm and 1.0 × 10^−7^ atm respectively). ***γ***-LiAlO_2_ phase appears at 0.005 atm and there is a complete transformation to ***γ***-LiAlO_2_ at 1.0 × 10^−7^ atm. Here, when CO_2_ partial pressure is at the lowest level the particle growth increases rapidly confirming the lithium aluminate instability, whereas at higher values the morphology remains quite unchanged showing a beneficial effect of CO_2_.

### 4.2 Particle coarsening and phase transition

The particle coarsening and the phase transformation are two inter-linked processes, despite that the activation energy of particle growth is higher than the phase transition value [80]. Several studies have been performed on this topic, detecting different mechanisms as a function of working conditions [37,66,80,82]. Coarsening is driven by dissolution and precipitation processes. LiAlO_2_ can dissociate due to high working temperatures (Eq. (7)) or undergo a decomposition by reacting with oxygen ions (Eq. (9)) produced during the ionic dissociation of the carbonates under CO_2_ low partial pressures (Eq. (8)). The ions generated during LiAlO_2_ dissolution are redeposited on the big particle surface leading to the growth of a new phase large crystal, in other cases small crystals of a new phase are produced by deposition and then they agglomerate [37].

(7) $$LiAlO_{2}\;\to \;{Li}^{+}+{AlO}_{2}^{-}$$

(8) $$M_{2}CO_{3}\;\to \;{2M}^{+}+O^{2-}+CO_{2}\;\left(M=Li,Na\right)$$

(9) $$LiAlO_{2}+O^{2-}\to \;{Li}^{+}+{AlO}_{3}^{3-}$$

Referring to the equilibrium constant of the molten carbonate dissociation (Eq. (8)) [82], a superior stability of ***α***-LiAlO_2_ phase is confirmed at CO_2_ high partial pressures. In Ref. [80] a similar result was observed for ***γ***-LiAlO_2_ showing an accelerated particle growth in a CO_2_ poor environment. Following these studies, two coarsening mechanisms have been identified as a function of CO_2_ concentration: (i) Ostwald ripening at low values and (ii) growth by oriented attachment at high values. The first is a thermodynamically driven process that involves the dissolution of smaller particles and their agglomeration on the surface of larger ones, and it can explain the considerable large particle growth of LiAlO_2_ under basic melt conditions [66,82]. Here depending on the particle size variation there is a difference in LiAlO_2_ solubilities which develops a concentration gradient inducing the diffusion of the dissolved species from small particles to large particles, simultaneously favoring the phase transformation. For instance, in Ref. [82] Li_2_CO_3_ electrolyte decomposes at low CO_2_ partial pressures by forming Li_2_O and CO_2_ (Eq. (10)). Li_2_O and LiAlO_2_ produce then α-Li_5_AlO_4_ intermediate species (Eq. (11)) which reacts with surrounding CO_2_ giving the final ***γ***-LiAlO_2_ particles (Eq. (12)).

(10) $$2{Li}_{2}CO_{3}\;\to \;2{Li}_{2}O+2CO_{2}$$

(11) $$\alpha -LiAlO_{2}+2{Li}_{2}O\;\to \;{\alpha -Li}_{5}AlO_{4}$$

(12) $${\alpha -Li}_{5}AlO_{4}+2CO_{2}\to \;\gamma -LiAlO_{2}+{2Li}_{2}CO_{3}$$

However, in such a system both ***α*** or ***γ*** crystal nuclei could be formed by the dissolved species (i.e., Li^+^, AlO_2_^2−^, AlO_3_^3−^) in the carbonate melt, and here the final crystal structure depends on the interaction between these nuclei and the dissolved species.

At CO_2_ high concentration the main mechanism consists in the growth by oriented attachment where particles are spontaneously oriented toward crystalline facets coherent with those of adjacent particles to reduce the total surface energy. This orientation is less dependent on the solubility and the particle size distribution, different from the previously discussed process. The dissolved LiAlO_2_ allows the adjacent particles to move and rotate freely until they achieve a perfect lattice match [82].

As a possible solution, the degradation of LiAlO_2_ by particle growth and phase transition can be controlled using additives to adjust the basicity of the electrolyte melt, such as K_2_WO_4_ [80].

### 4.3 Chemical instability due to reinforcing agents

Reinforcing agents have a fundamental role to avoid the cell mechanical instability. If on one hand they increase the lifetime reducing the crack formation risk, on the other hand they cause material changes reacting with the carbonate-based electrolytes. Indeed, commonly used Al reinforcement reacts with lithium carbonates (Eq. (13)) provoking the electrolyte redistribution due to the change in Li/K eutectic composition by the Li ion consumption. Here it results in an increase of the cell internal resistance.

(13) $$2Al+{Li}_{2}{CO}_{3}+\frac{3}{2}O_{2}\to 2LiAlO_{2}+{CO}_{2}$$

Li containing precursors (i.e., formate, acetate, etc.) have to be introduced within the matrix to avoid Li-ion shortage and minimize electrolyte losses [25]. For instance, in case of Al reinforcing particles [39], the cell durability can be increased by adding Li_2_CO_3_ particles as a further Li ^+^ ion source to have a compensation effect. However, looking at experimental tests, a performance degradation is still present after 1000 working hours due to Li ^+^ shortage issues since the Li_2_CO_3_ particles remain mainly in solid phase at MCFC operating temperature (∼650 °C) due to a melting point of ∼720 °C without here enriching the electrolyte. Alternatives to Li_2_CO_3_ powders directly incorporated into the matrix are: i) Li_2_CO_3_ addition in the cathode assuming its following migration to the matrix and ii) LiOH storage in the cathode channel of the separator. In the first option, the samples show a stable performance for only quite short periods since Li_2_CO_3_ added in cathode cannot reach the matrix facing with the same issue underlined before. Considering the second case, the LiOH introduction results more suitable since all cell components are porous at LiOH melting point (∼462 °C) due to the already occurred burning out of the additives and so it can easily penetrate through the cathode and cover Al particles in the matrix. Therefore, before the electrolyte eutectic mixture melting (∼500 °C), LiOH reacts with Al, as shown in Eq. (14), avoiding the interaction between Al particles and Li_2_CO_3_ during cell operation [39,85]. Li–Al particle reaction can form ***α***-LiAlO_2_ phase that increases the wettability of the matrix, not only preventing the electrolyte deterioration but also prolonging the reinforcement effects of Al on the matrix strength.

(14) $$2Al+2LiOH+\frac{3}{2}O_{2}\to 2LiAlO_{2}+H_{2}O$$

Undesired lithiation reactions can take place also in case of alumina-based fibers (Eq. (15)) and high performing Al-foam based matrixes, requiring further Li sources to guarantee a sufficient availability of the electrolyte [36,79].

(15) $${Al}_{2}O_{3}+{Li}_{2}{CO}_{3}\to 2LiAlO_{2}+{CO}_{2}$$

## 5 MCFC matrix: composite electrolyte

Composite electrolytes have been proposed as an alternative matrix structure aiming at the cell conductivity enhancement [86]. They consist in a porous anionic conductive ceramic matrix which hosts alkaline carbonate mixtures. These materials were firstly applied in SOFC (Solid Oxide Fuel Cell) technology to reduce the working temperatures without worsening the cell performance due to the ohmic resistance increase [87,88]. However, as a function of the reaction environment, this configuration is a hybrid SOFC-MCFC cell. MCFC operation results the most favorable since CO_2_ presence is beneficial to preserve the carbonates which could hydrogenate due to the steam at the anodic side forming hydroxides and decompose to alkaline oxides producing CO_2_ [89,90].

**Fig. 4:**
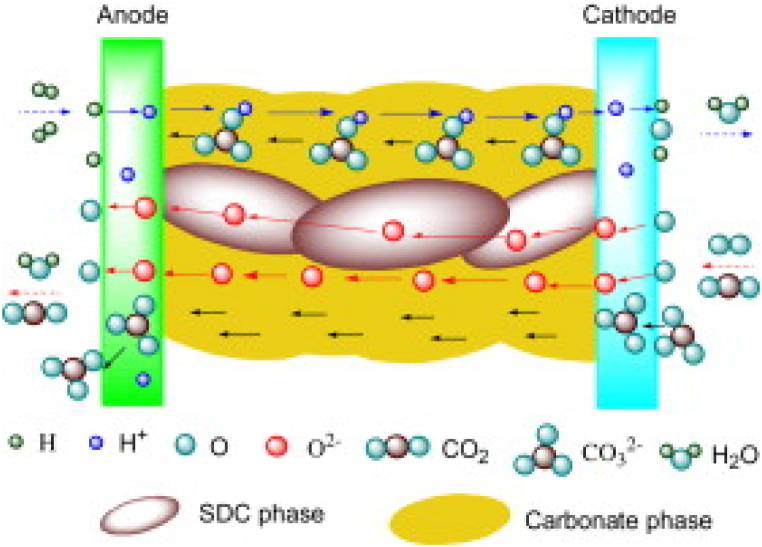
Ionic paths through composite electrolyte based on anionic conductive oxides impregnated by molten carbonates [99].

The most suitable ceramic material consists of ceria due to its good stability with molten carbonates. Both pure ceria and doped oxides, such as Samarium Doped Ceria (SDC) and Gadolinium Doped Ceria (GDC) have been proposed; however, the last is the less performing since Gd oxides can react with carbonates resulting in cation exchanges [91]. More recent studies have suggested the use of YSZ (Yttrium Stabilized Zirconia) which is the most established oxygen conductor for SOFC technology but it shows an inferior ionic conductivity at lower temperatures [85,92,93]. Eutectic binary or ternary mixtures are commonly applied as the molten phase [94]. In such a composite electrolyte, two parallel reaction paths occur: (i) O^2−^ conduction mainly within the ceramic materials and (ii) CO_3_^2−^ conduction through the molten carbonate bulk from cathodic to anodic side if the percolation threshold fraction is overcome reaching 30–40 vol% [87]. At low fed CO_2_, molten carbonates can also conduce oxygen due to CO_3_^2−^ weak dissociation in the salt phase [95]. Under a H_2_ rich atmosphere, this structure is characterized by a ternary conduction involving H^+^ migration from anode to cathode by a HCO_3_^−^ transition state (Fig. 4), as demonstrated by the water presence at both cell sides [96]. However, a single path usually prevails depending on the applied conditions. At common working temperatures the anionic based conduction mechanisms have the main weight, whereas between 200 and 600 °C the protonic one provides the highest contribution detecting almost all produced water at the cathode [97]. Coupling ceria and carbonates not only increases the ionic conductivity of the electrolyte layer, but also reduces the ceramic electronic conductivity creating a core-shell structure where a carbonate continuous layer on the ceria suppresses Ce^4+^ to Ce^3+^ reduction [90,98].

The traditional manufacturing procedure consists of the solid-state method, where ceria and carbonates powders are mixed to have a homogeneous distribution within the composite electrolyte. Nevertheless, this technique does not produce a very stable structure since the high sintering temperature required by ceria production cannot be reached to avoid the carbonate evaporation. Carbonate infiltration after ceramic sintering is a valid alternative which allows for a high density, mechanical strength, conductivity and thermal stability [100,101], but makes the loading control very difficult [102]. Nano ceria particles within composite electrolytes have been also tested with the aim of increasing the conductivity and the lifetime [103,104]. Indeed, a “superionic highway” at the ceramic-carbonate boundary has been supposed due to the formed space-charge layer resulting in a high concentration of defects and a fast ion migration [105,106]. Table 6 compares several tested composite electrolytes knowing that the target conductivity value is above 0.1 S/cm at 600 °C.

**Table 6:** Composite electrolyte conductivity and cell peak power density at variable working temperatures.

Reference	Material	Temperature (°C)	Composite electrolyte conductivity (S/cm)	Peak power density (mW/cm^2^)
[85]	10YSZ with (Li,K)_2_CO_3_	650	–	117
[90]	GYDC with (Li,Na)_2_CO_3_	550	0.260	520
[91]	8YSZ with (Li,Na)_2_CO_3_	600	0.002	–
[92]	8YSZ with (Li,K)_2_CO_3_	650	–	4
[93]	SDC with (Li,Na,K)_2_CO_3_	600	0.10–0.20	–
[100]	GSZ with (Li,Na)_2_CO_3_	600	0.500	–
[101]	GDC with (Li,Na)_2_CO_3_	600	0.150	150
[105]	8YSZ with (Li,Na)_2_CO_3_	650	0.003	–
[107]	GDC with (Li,K)_2_CO_3_	600	0.115	–
[108]	SrCe_0.6_Zr_0.3_Er_0.1_O_3-δ_ with (Li,Na)_2_CO_3_	600	0.140	247
[109]	SrCe_0.6_Zr_0.3_Lu_0. 1_O_3-δ_ with (Li,Na)_2_CO_3_	600	0.086	255
[110]	CeO_2_ with (Li,Na,K)_2_CO_3_	550	0.300–0.200	910

## 6 Conclusions

The molten carbonate fuel cell operation is based on the fuel electrochemical oxidation by carbonate ions migrating in the molten salts retained in a ceramic porous structure defined as matrix. Here its features in terms of the electrolyte wettability and the mechanical strength have significant effects on the cell performance and stability. Referring to previous studies, the main achievements are listed below.

• the state-of-the-art material consists of the inert lithium aluminate (LiAlO_2_) strengthened by Al based reinforcement agents as particle, fibers and foam;
• two LiAlO_2_ allotropic forms differ as the *α*-phase stable at low temperatures and CO_2_ rich atmospheres, the *γ*-phase stable at high temperatures and H_2_ rich atmospheres;
• the tape casting with organic solvents and the layer deposition are the main forming techniques for the planar and the tubular design respectively;
• an alternative solution is based on the composite electrolyte use as anionic conductive ceramic matrixes (i.e., fluorites) to exploit both CO_3_^2−^ and O^2−^ ion migration.

Several criticalities characterize the state-of-the-art lithium aluminate matrixes and the composite electrolyte structures in view of still several issues on the effective available power, the system stability and aging. Despite of some on-going megawatt scale plants, the attended lifetime of lithium aluminate-based cells cannot permit a competitive scenario of up to seven working years. Referring to the matrix degradation, the only partially resolved point consists of the mechanical instability by increasing the matrix strength but simultaneously leading to the electrolyte loss risk. LiAlO_2_ phase transition and particle coarsening are still almost unsolved, causing the structural change and the crack formation. Moreover, lithium aluminate manufacturing should be updated to face with environmental issues by introducing more eco-friendly procedures and avoiding toxic substances as solvents (note that matrixes derived from the aqueous tape casting are not comparable with traditional ones). In view of these severe penalizations, some alternatives have been proposed in terms of both cell geometry and matrix materials, nevertheless they have a technological readiness level too low to result feasible at a commercial level. The tubular configuration performance has not yet well established, lacking completely a detailed microstructural and electrochemical characterization. More research works were devoted to composite electrolyte applications, but the knowledge on their nominal operation and aging is very approximated under MCFC real working conditions.

Here further studies would aim at the optimization of matrix material and microstructure as well as an effective manufacturing protocol to favor the technology scale-up, requiring the coupling of experimental tests and modelling to have a deep knowledge of all involved phenomena.

## Data availability statement

Data was not deposited into a public repository since all data essential for understanding the study are already present into the manuscripts or the references where they can be found are clearly specified.

## CRediT authorship contribution statement

**Asrar A. Sheikh:** Conceptualization, Data curation, Investigation, Methodology, Resources, Writing – original draft. **Fiammetta R. Bianchi:** Data curation, Formal analysis, Writing – original draft, Writing – review & editing, Methodology, Supervision. **Dario Bove:** Formal analysis, Methodology, Supervision, Writing – original draft, Writing – review & editing, Investigation. **Barbara Bosio:** Conceptualization, Formal analysis, Methodology, Supervision, Validation.

## Declaration of competing interest

The authors declare that they have no known competing financial interests or personal relationships that could have appeared to influence the work reported in this paper.
